# Customizing Computerized Adaptive Test Stopping Rules for Clinical Settings Using the Negative Affect Subdomain of the NIH Toolbox Emotion Battery: Simulation Study

**DOI:** 10.2196/60215

**Published:** 2025-03-21

**Authors:** Saki Amagai, Aaron J Kaat, Rina S Fox, Emily H Ho, Sarah Pila, Michael A Kallen, Benjamin D Schalet, Cindy J Nowinski, Richard C Gershon

**Affiliations:** 1 Department of Medical Social Sciences, Northwestern University Feinberg School of Medicine, Chicago, IL, United States,; 2 College of Nursing, University of Arizona, Tuscon, AZ, United States; 3 Department of Epidemiology and Data Science, Amsterdam University Medical Centers, Amsterdam, The Netherlands

**Keywords:** computerized adaptive testing, CAT, stopping rules, NIH Toolbox, reliability, test burden, clinical setting, patient-reported outcome, clinician

## Abstract

**Background:**

Patient-reported outcome measures are crucial for informed medical decisions and evaluating treatments. However, they can be burdensome for patients and sometimes lack the reliability clinicians need for clear clinical interpretations.

**Objective:**

We aimed to assess the extent to which applying alternative stopping rules can increase reliability for clinical use while minimizing the burden of computerized adaptive tests (CATs).

**Methods:**

CAT simulations were conducted on 3 adult item banks in the NIH Toolbox for Assessment of Neurological and Behavioral Function Emotion Battery; the item banks were in the Negative Affect subdomain (ie, Anger Affect, Fear Affect, and Sadness) and contained at least 8 items. In the originally applied NIH Toolbox CAT stopping rules, the CAT was stopped if the score SE reached <0.3 before 12 items were administered. We first contrasted this with a SE-change rule in a planned simulation analysis. We then contrasted the original rules with fixed-length CATs (4‐12 items), a reduction of the maximum number of items to 8, and other modifications in post hoc analyses. Burden was measured by the number of items administered per simulation, precision by the percentage of assessments yielding reliability cutoffs (0.85, 0.90, and 0.95), and accurate score recovery by the root mean squared error between the generating θ and the CAT-estimated “expected a posteriori”–based θ.

**Results:**

In general, relative to the original rules, the alternative stopping rules slightly decreased burden while also increasing the proportion of assessments achieving high reliability for the adult banks; however, the SE-change rule and fixed-length CATs with 8 or fewer items also notably increased assessments yielding reliability <0.85. Among the alternative rules explored, the reduced maximum stopping rule best balanced precision and parsimony, presenting another option beyond the original rules.

**Conclusions:**

Our findings demonstrate the challenges in attempting to reduce test burden while also achieving score precision for clinical use. Stopping rules should be modified in accordance with the context of the study population and the purpose of the study.

## Introduction

### Background

The patient’s perspective, typically collected using patient-reported outcome measures (PROMs), is an imperative component of effective medical decision-making and treatment evaluation [1-4]. However, PROMs can be time-consuming for respondents to complete and difficult for clinicians to interpret, thus limiting their practical utility. Increasing importance has therefore been placed on minimizing response burden. For example, newer PROM initiatives have incorporated item response theory (IRT) and computerized adaptive test (CAT) capabilities, which enable the derivation of efficient, reliable, and precise assessments from large banks of items [5,6].

The NIH Toolbox for Assessment of Neurological and Behavioral Function is one such measurement initiative [7]. The NIH Toolbox is a set of neurobehavioral measures that can be used to assess sensory, motor, emotional, and cognitive functioning among individuals aged 3 to 85 years. While many measures included in the NIH Toolbox focus on performance-based assessments, the Emotion Battery consists exclusively of PROMs assessing emotions such as sorrow, joy, or fear [8]. The battery consists of 4 subdomains: Negative Affect (Anger, Fear, and Sadness), Psychological Well-Being (Life Satisfaction, Meaning and Purpose, and Positive Affect), Social Relationships (Companionship, Fear, Perceived Social Support, Positive Social Development, and Social Distress), and Stress and Self-Efficacy (Self-Efficacy and Stress). Across all item banks, higher scores indicate higher levels of the construct being assessed (eg, a higher score on the Sadness measure indicates higher levels of sorrowful feelings or emotions) [8]. The item banks were calibrated using the graded response IRT model [9]. This calibration lines up all the items along a common mathematical metric, which allows any subset of items to be used to track a given patient’s performance longitudinally or compare performance scores across patients under a common metric [10-12]. The NIH Toolbox Emotion Battery calculates “expected a posteriori” (EAP) scores, which are a type of IRT-based score, on the θ metric (mean 0, SD 1). These scores are then transformed to a *t* score metric (mean 50, SD 10) for individual reporting. The Emotion Battery provides a psychometrically strong option for evaluating emotional health that is particularly well suited for use in combination with other performance-based measures included in the NIH Toolbox.

Measures in the NIH Toolbox Emotion Battery are administered as fixed forms or variable-length CATs. The fixed forms consist of a set of items that are all administered in the same order during every administration. Conversely, when variable-length CATs are administered, the first item typically reflects average functioning or symptom severity, as the maximum weighted information value for a starting item is at the population mean (θ=0; *t* score=50). The response to that item is then used to calculate the provisional score estimate for the measured construct and to identify the best “next” item, within the remaining items in the bank, that will maximize weighted information based on the provisional ability estimate for the patient [13-15]. A patient’s estimated θ score is iteratively refined after each item until (1) a minimum number of items is administered and either (2a) a prespecified level of measurement precision is achieved or (2b) a prespecified maximum number of items is administered. These conditions are known as “stopping rules” [16,17]. After 4 items have been administered (the minimum), the default for the NIH Toolbox CAT administration is to stop the CAT after either 12 items have been administered (the maximum) or the SE of the EAP score estimate is less than 0.3, which corresponds to a minimum reliability of 0.91. Other CATs may implement these or additional stopping rules, or may include content balancing or item exposure constraints. There is also a fully Bayesian approach for estimating ability using the Markov chain Monte Carlo method, which can be used to incorporate empirical information about candidates [18]. The use of empirical prior information has been shown to reduce response burden for patients and clinical practitioners [19]. While these other stopping rules may appear in other contexts (including in the NIH Toolbox Cognitive Battery for exposure constraints), the NIH Toolbox Emotion CATs do not implement any of these other features [20].

Compared to legacy measures, these stopping rules substantially reduce response burden, yet NIH Toolbox CATs still impose a significant burden on respondents with no or minimal symptoms. Similar issues have been documented in educational measurement using precision rules as a primary criterion for stopping rules [21]. For example, if an item bank has numerous items reflecting extreme sadness but few items reflecting minimal sadness, the statistically most-informative items for someone experiencing little-to-no sadness will be exhausted quickly, and additional items will be increasingly less targeted and unable to notably improve the score precision (ie, reducing the SE).

In such a scenario, the CAT will continue to select the next best (but still mistargeted) item until the maximum CAT length is reached. While there are sufficient items in each item bank to stratify pathology, if a person is not impaired with respect to the domain being explored, it is difficult or impossible to accurately differentiate how healthy a person is (eg, how not sad a person may be). As a result, patients with the best health in a given domain are more likely to be administered the maximum 12 items in a CAT as compared to patients experiencing more severe symptoms [22]. Therefore, the first goal of this study was to develop and systematically assess alternative stopping rules that would minimize response burden and frustration for these patients with few-to-no symptoms, as patients with wide range of levels of functioning are administered NIH Toolbox measures.

The second goal of this study was to alter stopping rules to improve reliability and, more specifically, to achieve clinically relevant reliability (eg, reliability ≥0.95). This is especially important today as the NIH Toolbox is increasingly being used with clinical populations and in clinical settings, even though it was developed specifically for use in research. As stated, the current CAT stopping rules call for a test to end when the SE is <0.3, which corresponds to a minimum reliability of 0.91. Although this level of reliability is recommended for use in general populations [23], it may be inadequate to identify clinically relevant symptoms and individual patient changes across time [24]. Thus, as NIH Toolbox measures are implemented more frequently for clinical use in health care delivery settings [25,26], the ability to achieve this level of reliability appears increasingly valuable. Reaching clinical-level reliability will lead to fewer errors in patient assessments and increase confidence in the treatments or type of care to be recommended.

Recent work has evaluated alternative approaches to CAT administration in an effort to promote more efficient and effective testing. In their simulation study, Kallen and colleagues [22] shortened the minimum test length for individuals completely denying symptoms to only 2 items and added a minimum SE-change rule, while retaining the SE <0.3 rule and a maximum test length of 12 items. The minimum SE-change rule allowed the CAT to stop when the CAT algorithm detected that the administration of additional items would not reduce the SE. The impact of adding the SE-change rule was negligible. This simulation, combined with the shortened test length for individuals reporting absolutely no symptoms, addressed the concern that high-functioning individuals often receive lengthier CATs under the originally applied stopping rules for the NIH Toolbox. Importantly, this approach yielded scores that varied minimally from those obtained using the original stopping rules, particularly for scores in the clinically relevant range. Very few administrations required more than 8 items under these new stopping rules. However, the application of a SE <0.3 stopping rule retained a target reliability of 0.91, below a clinically relevant range (eg, ≥0.95), thus questioning the appropriateness of these rules as optimal for application in clinical settings.

### Purpose of This Study

The NIH Toolbox has been increasingly used in real-world clinical settings, and patients with a wide range of levels of functioning complete these measures. The purpose of this study was to propose and evaluate new stopping rules that could optimally balance two competing priorities and goals that are increasingly common in both research and clinical practice:

1. Increase reliability (≥0.95) for the clinical population (ie, adjust the SE threshold to require higher reliability) without unacceptably increasing the number of items
2. Decrease response burden for nonsymptomatic individuals (ie, reduce the number of items administered) without unacceptably reducing reliability (<0.85)

By using in-person, in-production item banks, this study provides real-world insights that go beyond the theoretical understanding of how stopping rules function.

## Methods

### Planned Simulation Study

#### Design

Firestar was used to simulate examinee responses and item bank CAT administration [27]. Three adult (age ≥18 years) NIH Toolbox Negative Affect item banks (Anger Affect, Fear Affect, and Sadness) were evaluated. All 3 of the item banks contain at least 8 items and are IRT-calibrated and EAP-scored (Table 1). More detailed descriptions of the item banks, along with examples, can be found in Table S1 in Multimedia Appendix 1. The simulation study manipulated the CAT administration to implement both the original stopping rules and a new set of rules, which we named the “SE-change” rules. The SE-change rules terminated a test when (1) a minimum of 4 items or maximum of 8 items had been administered, thus decreasing the maximum test length; (2) SE was <0.224, thus increasing target reliability to 0.95; or (3) the SE was reduced by less than 0.01, thus stopping the test when measurement precision would not increase sufficiently by administering an additional item (Table 2). We hypothesized that the SE-change stopping rules would increase the proportion of assessments achieving clinically relevant reliability without unacceptably increasing the number of items administered to nonsymptomatic individuals.

**Table 1.** NIH Toolbox Emotion Battery measures from the Negative Affect subdomain included in the simulation.

Measure	Typical computer adaptive test administration (number of items available in bank)	Slope parameter, mean (SD)	Average within-item thresholds, mean (SD)
Anger Affect	22	2.22 (0.43)	1.45 (0.44)
Fear Affect	29	2.72 (0.78)	1.44 (0.40)
Sadness	28	3.14 (0.67)	1.27 (0.24)

**Table 2.** Stopping rule models evaluated.

Stopping rule model	Minimum items	Maximum items	SE threshold for interim stopping rule	SE-change threshold for interim stopping
Original	4	12	0.3 (reliability 0.91)	—^a^
SE-change	4	8	0.224 (reliability 0.95)	<0.01
4-item fixed	4	4	—	—
5-item fixed	5	5	—	—
6-item fixed	6	6	—	—
7-item fixed	7	7	—	—
8-item fixed	8	8	—	—
9-item fixed	9	9	—	—
10-item fixed	10	10	—	—
11-item fixed	11	11	—	—
12-item fixed	12	12	—	—
Reduced maximum	4	8	0.224 (reliability 0.95)	—

a Not applicable.

Interim item selection (using maximum posterior weighted information), starting item, and scoring (using EAP estimates with a standard normal prior) were constrained to be the same across all simulated CATs. For each stopping rule, 1000 responses were simulated with uniform distribution (for θ values from –4 to 4) using the full item banks. The simulations used a uniform distribution to eliminate artificial biases that could be inadvertently introduced if the person ability distribution was simulated such that it mimicked the item difficulty distribution. The unit of analysis was individual CAT assessments. The CAT simulations proceeded until one of the stopping rules was met. All simulations and analyses were conducted using the *Firestar* package [27] and R (version 3.6.3; R Foundation for Statistical Computing).

#### Evaluation Criteria

We used 4 evaluation criteria—precision, accurate score recovery, test burden, and efficiency—to compare the impact of the different stopping rules. (1) Precision was evaluated by exploring the percentages of assessments yielding reliabilities less than 0.85, between 0.85 and 0.90, between 0.90 and 0.95, and greater than 0.95 across stopping rules. This approach allowed us to compare the reliability achieved by each set of stopping rules in a more nuanced way rather than assessing mean reliability, which can obscure the impact of stopping rules on conditional reliability across the trait range. Additionally, maximum attainable information curves were generated to determine theoretically attainable reliability for fixed-length CATs with up to 12 items for each of the 3 item banks. (2) Accurate score recovery refers to an evaluation of whether the CAT was able to recover the generating score and index the variability in score recovery; for this, we calculated the root mean squared error (RMSE) between the generating θ and the CAT-estimated, EAP-based θ from each version of the CAT. (3) Test burden, or response burden, was evaluated by comparing the number of items administered as a function of each set of stopping rules. (4) Efficiency was defined as the Fisher information per number of items administered, which is equivalent to (1/SE^2^)/item count, such that higher values represent greater efficiency.

### Post Hoc Simulation Study

Achieving reliability ≥0.85 is considered a critical issue for the priority of increasing reliability. However, the reduced number of items in the SE-change stopping rule led to an increase in the assessments achieving low empirical reliability (<0.85). That is, assessments that had higher reliability (>0.85) under the original stopping rules were no longer able to maintain the same level of precision under the SE-change rules. As one of our two goals was to reduce burden without unacceptably reducing reliability, we conducted post hoc analyses in which we evaluated two new sets of stopping rules to more optimally balance the two goals: increasing reliability for the clinical population while decreasing burden for nonsymptomatic individuals.

All methods used in the planned simulations, as outlined, were replicated for this post hoc analysis; the only adjustments made were the addition of two new sets of stopping rules: (1) fixed-length CATs ranging in length from 4 to 12 items, and (2) “reduced maximum” stopping rules in which tests were terminated when SE was <0.224 or a maximum of 8 items had been administered (Table 2). The same evaluation criteria were used to compare the different stopping rules.

### Ethical Considerations

The research presented herein was a simulation study, which was determined to not be human subjects research. Therefore, ethical review and approval was not necessary. This study was conducted using data from the NIH Toolbox Negative Affect item banks. The analysis did not involve identified patient information or any interaction with human subjects. As such, it did not meet the criteria for human subjects research and did not require ethics board review.

## Results

### SE-Change Stopping Rules vs Original Stopping Rules

The average percentage of simulated assessments yielding reliability at cutoffs of 0.85, 0.90, and 0.95 across stopping rules can be found in Table 3. For Fear Affect and Sadness, a larger percentage of assessments yielded reliability >0.95 per the SE-change stopping rules compared to the original stopping rules (+33.9% Fear Affect;+20.4% Sadness). For Anger Affect, neither set of stopping rules were able to yield the clinically relevant target reliability of 0.95. This result is because Anger Affect is a “weaker” bank (ie, the maximum attainable test information function is lower) that requires more items to be administered in order to distinguish different levels of anger and to reach 0.95 reliability. Most importantly, the percentage of assessments yielding low reliability for all three item banks (ie, <0.85) substantially increased (+2.7% Anger Affect;+4.7% Fear Affect;+3.4% Sadness).

**Table 3.** Proportion of simulated assessments meeting various levels of reliability.

Domain and level of reliability		Proportion of assessments meeting specified level of reliability (%)											
		Original	SE-change	4-item, fixed	5-item, fixed	6-item, fixed	7-item, fixed	8-item, fixed	9-item, fixed	10-item, fixed	11-item, fixed	12-item, fixed	Reduced maximum
**Anger Affect**													
	<0.85	27.4	30.1	43.4	34.2	30.9	30.8	30	29.6	28.9	28.8	28.6	28.6
	0.85-0.90	5.5	12.8	56.6	52.7	17.7	11.3	8.7	6.7	6.4	5.1	4.9	6.9
	0.90-0.95	67.1	57.1	0	13.1	51.4	57.9	61.3	63.7	64.7	66.1	47.4	64.5
	≥0.95	0	0	0	0	0	0	0	0	0	0	19.1	0
**Fear Affect**													
	<0.85	31.5	36.2	36.6	36.4	35.2	35.2	35	33.8	33	32.9	32.6	33.5
	0.85-0.90	3.5	5.7	15.7	12.9	9.2	8.1	7.2	7	6.2	5.1	5	4.9
	0.90-0.95	65	24.2	47.7	50.7	25.3	20	15.8	14.2	15.1	15.6	14.2	21.8
	≥0.95	0	33.9	0	0	30.3	36.7	42	45	45.7	46.4	48.2	39.8
**Sadness**													
	<0.85	32.5	35.9	37.8	49.8	36.4	36.1	35.6	35.6	35.1	34.9	34.7	36.1
	0.85-0.90	5.3	18.3	19.9	4.4	16	12.8	10.2	8.9	8	7.6	6.2	5.8
	0.90-0.95	49.1	12.4	41.3	15.6	13.9	12.7	13.8	13.6	13.6	13.2	14.1	17.9
	≥0.95	13.1	33.4	1	30.2	33.7	38.4	40.4	41.9	43.3	44.3	45	40.2

**Figure 1.**
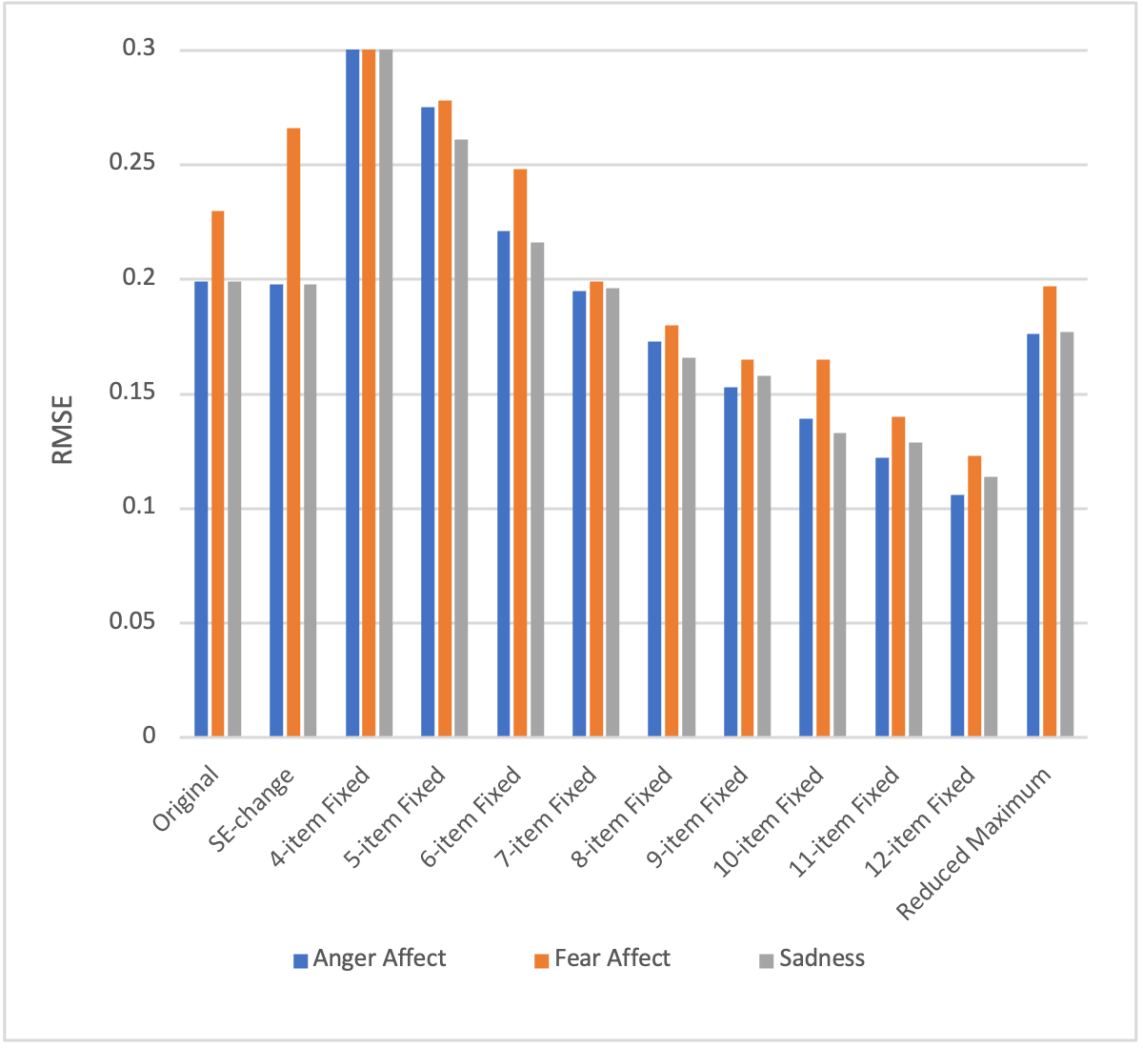
Root mean squared error (RMSE) of θ estimates.

Concerning accurate score recovery, the RMSE of θ estimates are presented in Figure S1 in Multimedia Appendix 2 and Figure 1, respectively. The RMSE increased for Fear Affect (+0.036), while it decreased slightly for the other two banks (Anger Affect –0.001; Sadness –0.001). Test burden decreased for all banks per the SE-change rules as opposed to the original stopping rules, as shown in Table 4 (Anger Affect −1.26 items; Fear Affect −1.39 items; Sadness −2.64 items). In the original stopping rules, the average number of items administered was 7.9, compared to 6.1 items under the SE-change stopping rules, a 22% savings in response burden across banks (Table 4). The largest decrease in response burden observed was 33% for the Sadness bank.

**Table 4.** Average number of items administered among 1000 simulated assessments.

Item bank	Number of items in item bank	Stopping rules		
		Original	SE-change	Reduced Maximum
Anger Affect	22	8.33	7.07	8.00
Fear Affect	29	7.40	6.01	7.36
Sadness	28	7.83	5.19	6.86

### Fixed-Length CATs vs Original Stopping Rules

The fixed-length stopping rules led to an increased percentage of assessments reaching >0.85 reliability, as the test length is increased relative to the original stopping rules (Table 3). Per the fixed-length stopping rules, the target reliability level of 0.95 was achieved in the simulated assessments by only administering 6 items for Fear Affect and 4 items for Sadness, which is shorter than the average test length using the original stopping rules. However, more notably, the fixed-length CATs with 8 items or fewer also increased in simulated assessments with reliability <0.85 relative to the original stopping rules. Anger Affect was an exception and required 12 items to have any simulated assessments achieve the target reliability, and the assessments with reliability <0.85 did not improve relative to the original stopping rules even when administering 12 items.

Figure S2 in Multimedia Appendix 3 illustrates the potential inefficiencies for fixed-length CATs with up to 12 items. Consistent with the simulated results, Figure S2 in Multimedia Appendix 3 shows that there could be notable differences in the number of items required to reach high reliability depending on the item bank. It is theoretically possible to achieve 0.95 reliability by administering at least 4 items for the Sadness and at least 5 items for the Fear Affect bank. Conversely, it can theoretically take at least 11 items for the Anger Affect bank to achieve 0.95 reliability, which suggests that the Anger Affect bank is a “weaker” bank that requires more items to be administered to distinguish levels of anger. Figure S2 in Multimedia Appendix 3 also suggests that individuals with little-to-no functioning of the trait (θ level=−1) will be subject to significant burden. For example, individuals with little-to-no sadness will never reach the 0.95 reliability threshold and will peak in maximum attainable information after 5 items, with diminishingly small gains in measurement precision. In this situation, high-functioning individuals will be administered more items until the maximum number of items is reached, thereby increasing burden.

The CAT was able to recover the generating score better when more items were administered in the fixed-length stopping rules as opposed to the original stopping rules. That is, RMSE of θ estimates decreased per the fixed-length stopping rules as opposed to the original stopping rules when administering more than 8 items for all banks. However, this improved accuracy score recovery came at a cost, as any fixed-length test greater than 8 items increases test burden relative to the original stopping rules (Table 4).

### Reduced Maximum Stopping Rules vs Original Stopping Rules

A larger percentage of assessments yielded reliability >0.95 per the reduced maximum stopping rules as opposed to the original stopping rules (+0% Anger Affect, +39.8% Fear Affect, +40.2% Sadness). Importantly, this change did not excessively increase the percentage of assessments achieving low reliability (+1.2% Anger Affect; +2.0% Fear Affect; +3.6% Sadness).

The reduced maximum stopping rules improved the accuracy score recovery. The generating θ scores and score estimates were strongly correlated (>0.99) across banks for both stopping rules. RMSE also decreased for all banks (Anger Affect –0.023; Fear Affect –0.033; Sadness –0.022). Additionally, the average number of items administered was 7.9 compared to 7.4 items under the reduced maximum, a 6% savings in response burden across banks (Table 4). The largest decrease in response burden observed was 12% for the Sadness bank. Figure S3 in Multimedia Appendix 4 illustrates that the reduced maximum stopping rule may not be the most efficient choice across the θ range, but in general, all the rules are broadly comparable across banks.

## Discussion

### Principal Findings

Measures within the NIH Toolbox Emotion Battery that are administered as CATs are programmed to terminate when prespecified stopping rules are achieved. While the originally applied stopping rules are effective for many test takers, nonsymptomatic individuals often are burdened with many questions that are not relevant to their current health status. Additionally, many of the item banks have sufficient statistical information to achieve clinical-level reliability, but the original stopping rules terminate the assessment before that level of reliability is met. With increasing use of the NIH Toolbox in clinical settings, it is critical to simultaneously balance competing priorities: (1) maximizing score precision for clinical use and (2) minimizing burden for nonsymptomatic individuals. We evaluated alternative stopping rules to achieve these two goals.

The SE-change stopping rules were effective at reducing overall burden and were effective at ensuring adequate reliability and score recovery. Perhaps most importantly, although the SE-change stopping rules increased the percentage of assessments achieving clinically relevant reliability (≥0.95), the percentage of assessments achieving low empirical reliability (<0.85) also increased to an unacceptable level. Ultimately, we determined that the detrimental impact of the SE-change stopping rules on the percentage of assessments achieving low empirical reliability outweighed the modest benefits observed regarding test length and the percentage achieving very high reliability. This finding prompted us to explore additional stopping rule paradigms in post hoc simulations.

The fixed-length stopping rules were also not effective. Although it was possible to reduce burden relative to the original stopping rules by administering fewer than 8 items, it came at the cost of inflating the percentage of assessments achieving low empirical reliability (<0.85) while worsening accurate score recovery. By administering 8 or more items, it was also possible to maximize assessments achieving high reliability (>0.95) but at the cost of increasing burden. Given the benefits of longer fixed-length CATs, we opted to explore the reduced maximum stopping rules to determine if we could maintain this improvement in reliability without requiring 8 items across all administrations of all banks.

This subsequent evaluation identified a paradigm that more effectively balanced precision and parsimony. In general, the reduced maximum stopping rules demonstrated the same increase in reliability obtained with the 8-item fixed-length CATs; however, these stopping rules allowed for further decreased burden, as 8 items were not always required to reach a reliability level of 0.95.

One exception to the observed trends was the Anger Affect item bank. In simulations, this bank was unable to achieve reliability >0.95 for the reduced maximum stopping rule, as greater than 11 items are theoretically required to reach the reliability level of 0.95. As Figure S2 in Multimedia Appendix 3 showed, Anger Affect is an example of a “weak” bank, in which more items are needed to be administered to obtain clinically relevant reliability. The strength or weakness of a bank is related to the magnitude of the item slope parameters and the (statistically related) test information function. The Anger slope parameters are lower—therefore they are less-strongly related to the latent construct and contain more “error variance” or noise in their ratings. One possible hypothesis is that there are numerous and varied reasons someone might endorse or not endorse an Anger item, so the construct as a whole is not as cleanly defined as sadness or fear. Individuals may engage in more self-monitoring about anger. Although Anger Affect will never achieve our clinically relevant target reliability with the reduced maximum stopping rule, it is not in our best interest to increase the test length to accommodate these “weaker” banks, as this will lead to increased response burden for all banks.

Given the competing priorities of improving reliability while minimizing burden, decisions for stopping rules should be adjusted depending on the context of the study population and the purpose of the study. The results of our study will be also relevant for other users who share a similar goal in achieving high reliability for use in clinical settings, as the NIH Toolbox has been increasingly used with clinical populations [28], while minimizing burden for nonsymptomatic individuals. When determining stopping rules, users should also consider the reliability achievable with the given item bank, the target reliability needed to answer their research or clinical questions, and the range of the domain in the sample population. Stopping rules should be modified after carefully considering these varied measurement contexts.

### Limitations

Findings are limited to the 3 NIH Toolbox Emotion Battery adult item banks studied. As this study was designed to be largely a statistical investigation, our planned and post hoc stopping rules only incorporated psychometric considerations. These stopping rules were not applied to identical response data, although all simulations were based on the same uniform population distribution. To avoid artificial biases, we conducted the analyses in a way that prevented the person ability distribution from being simulated to match the item difficulty distribution. However, this may restrict the generalizability of our findings to real-world person distributions [29,30]. Indeed, using SE stopping rules in a multidimensional CAT setting can reduce test length by upward of 33% [31-33].

We did not explore multidimensional CATs for this study because those assessments are built on the assumption that all measures within a given composite (eg, Negative Affect subdomain) are important and should be assessed. While we expect clinicians and researchers would want to use all measures within a given subdomain, we chose not to impose that requirement. Future studies could extend the analyses to other subdomains and PROMs by considering multidimensional adaptive testing and theoretically driven content balancing techniques. As this analysis only examined adult responses, future extensions of this work could also examine the Emotion Battery for a pediatric population.

### Conclusion

Taken together, these results demonstrate the challenges in attempting to reduce test burden while also achieving score precision for clinical use. The reduced maximum stopping rules provide one acceptable strategy to achieve these goals. The SE-change stopping rules and fixed-length stopping rules with less than 8 items are not recommended due to the sizable increase in simulations achieving empirical reliability <0.85. The reduced maximum stopping rules maximized the proportion of simulations achieving clinically relevant reliability (>0.95) without excessively inflating those achieving low reliability; they also decreased burden by allowing for fewer items to be administered when sufficient. That said, the reduced maximum stopping rules are not recommended for *all* occasions. Stopping rules should be modified in accordance with the context of the study population and the purpose of the study.

## Supplementary material

10.2196/60215 Multimedia Appendix 1 Description of item banks.

10.2196/60215 Multimedia Appendix 2 Correlation of the generating θ score with the estimated expected a posteriori score.

10.2196/60215 Multimedia Appendix 3 Maximum attainable information curves for Anger Affect, Fear Affect, and Sadness.

10.2196/60215 Multimedia Appendix 4 Comparisons of computerized adaptive test stopping rule efficiencies.

## Abbreviations

CAT: computerized adaptive test
EAP: expected a posteriori
IRT: item response theory
PROM: patient-reported outcome measure
RMSE: root mean squared error
