# Supplementary material for: Customizing Computerized Adaptive Test Stopping Rules for Clinical Settings Using the Negative Affect Subdomain of the NIH Toolbox Emotion Battery: Simulation Study
Source: JMIR Form Res. 2025 Mar 21;9:e60215. doi: 10.2196/60215 (PMC11951945; doi:10.2196/60215)
Supplement: Multimedia Appendix 1 [file formative-v9-e60215-s001.docx]

**Table S1** Description of item banks

| **Item bank** | **Description of item bank** | **Example item** |
| --- | --- | --- |
| **Anger-Affect** | Assessment of irritability, frustration, interpersonal sensitivity, envy, disagreeableness, and efforts to control anger. | In the past 7 days... I was irritated more than people knew.   1. Never 2. Rarely 3. Sometimes 4. Often 5. Always |
| **Fear-Affect** | Assessment of fear, anxious misery, hyperarousal, and somatic symptoms related to arousal | In the past 7 days... I felt fearful.   1. Never 2. Rarely 3. Sometimes 4. Often 5. Always |
| **Sadness** | Assessment of poor mood and negative perceptions of the self, the world, and the future | In the past 7 days... I felt helpless.   1. Never 2. Rarely 3. Sometimes 4. Often 5. Always |
