# Supplementary figures and images for: Customizing Computerized Adaptive Test Stopping Rules for Clinical Settings Using the Negative Affect Subdomain of the NIH Toolbox Emotion Battery: Simulation Study
Source: JMIR Form Res. 2025 Mar 21;9:e60215. doi: 10.2196/60215 (PMC11951945; doi:10.2196/60215)

Figure S1. *Correlation of the generating theta score with the estimated EAP score*


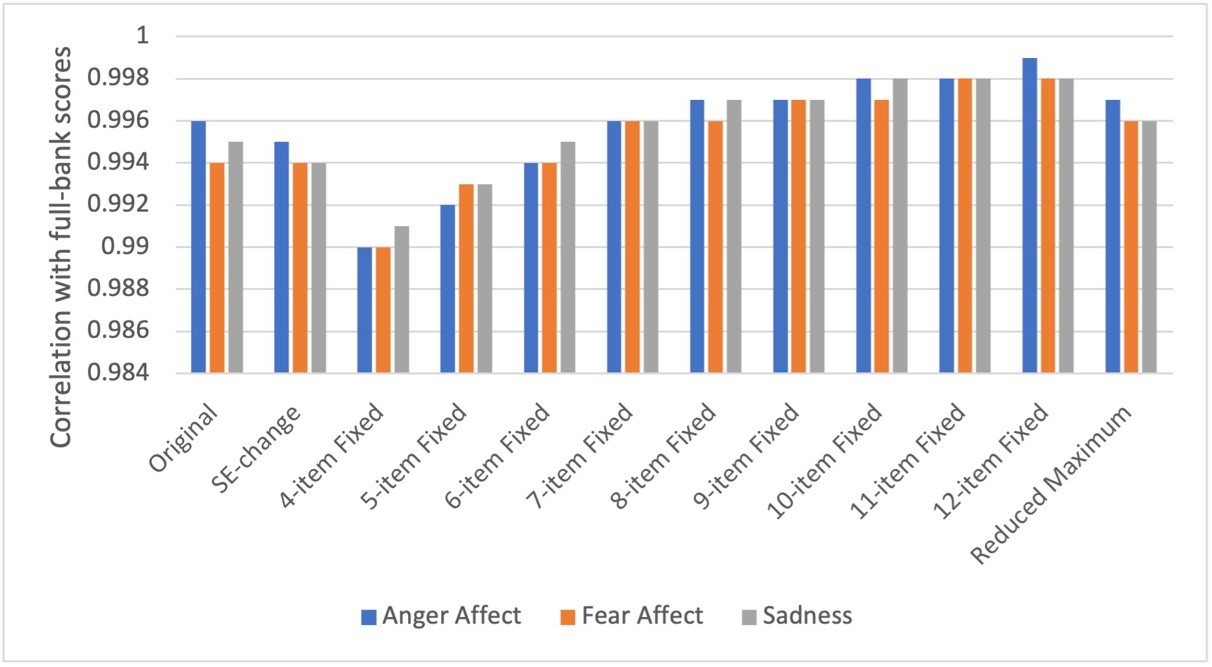

Supplement: Multimedia Appendix 2 [file formative-v9-e60215-s002.docx]

**Figure S3** Comparisons of CAT Stopping Rule Efficiencies

| 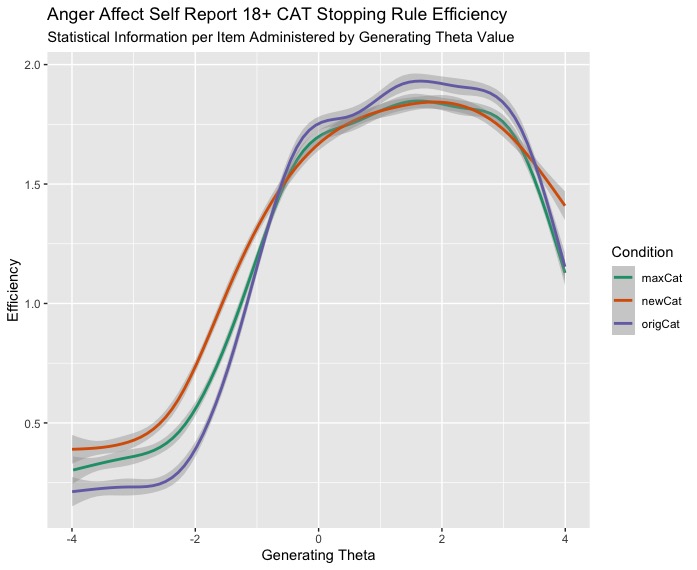 |
| --- |
| b)  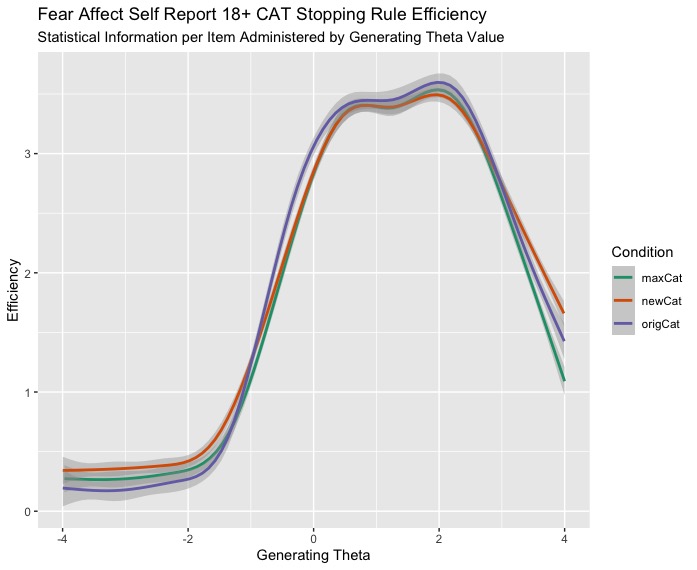 |
| c)  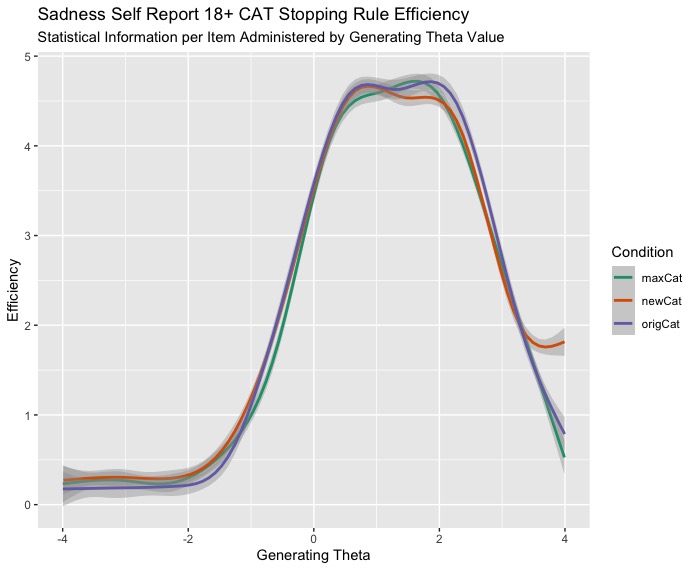 |

Supplement: Multimedia Appendix 4 [file formative-v9-e60215-s004.docx]
