# Supplementary material for: Customizing Computerized Adaptive Test Stopping Rules for Clinical Settings Using the Negative Affect Subdomain of the NIH Toolbox Emotion Battery: Simulation Study
Source: JMIR Form Res. 2025 Mar 21;9:e60215. doi: 10.2196/60215 (PMC11951945; doi:10.2196/60215)
Supplement: Multimedia Appendix 3 [file formative-v9-e60215-s003.docx]

Figure S2. *Maximum Attainable Information Curves for Anger Affect, Fear Affect, and Sadness*

| **a)** | 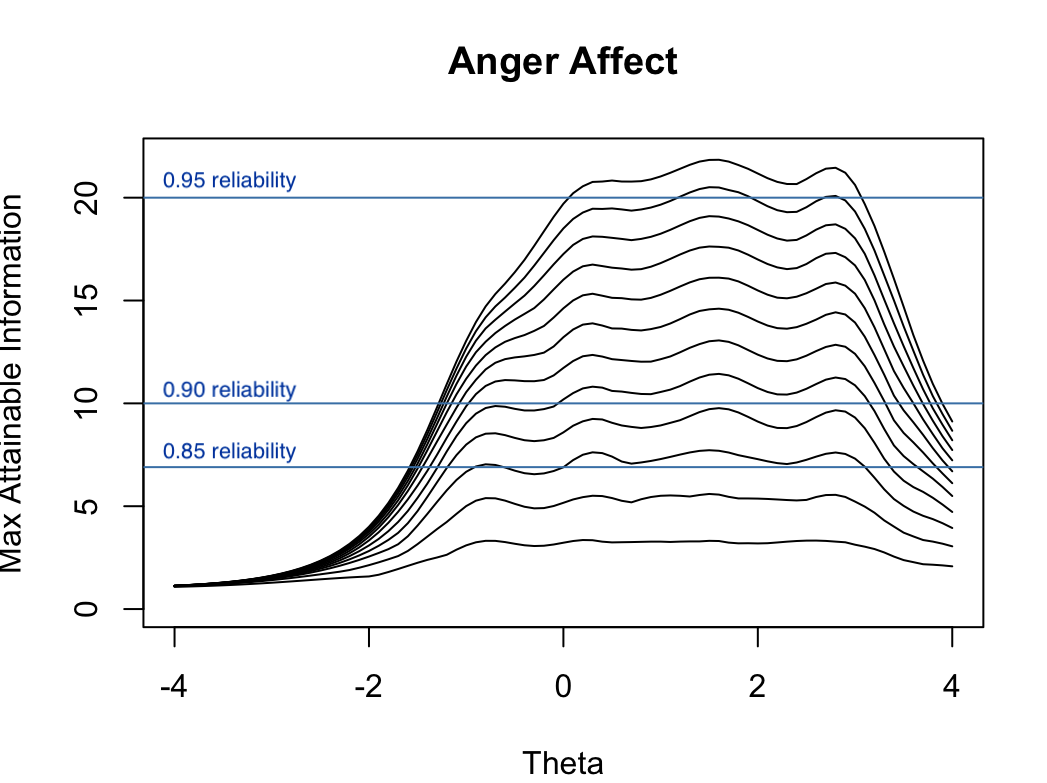 |
| --- | --- |
| b) | 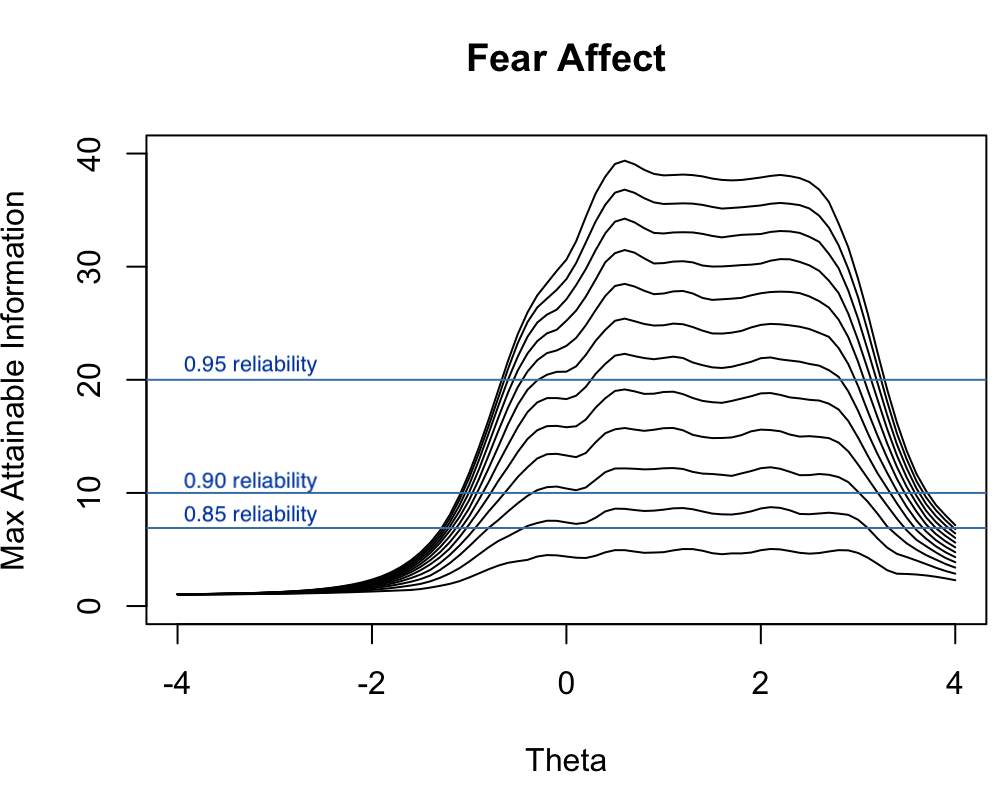 |
| c) | 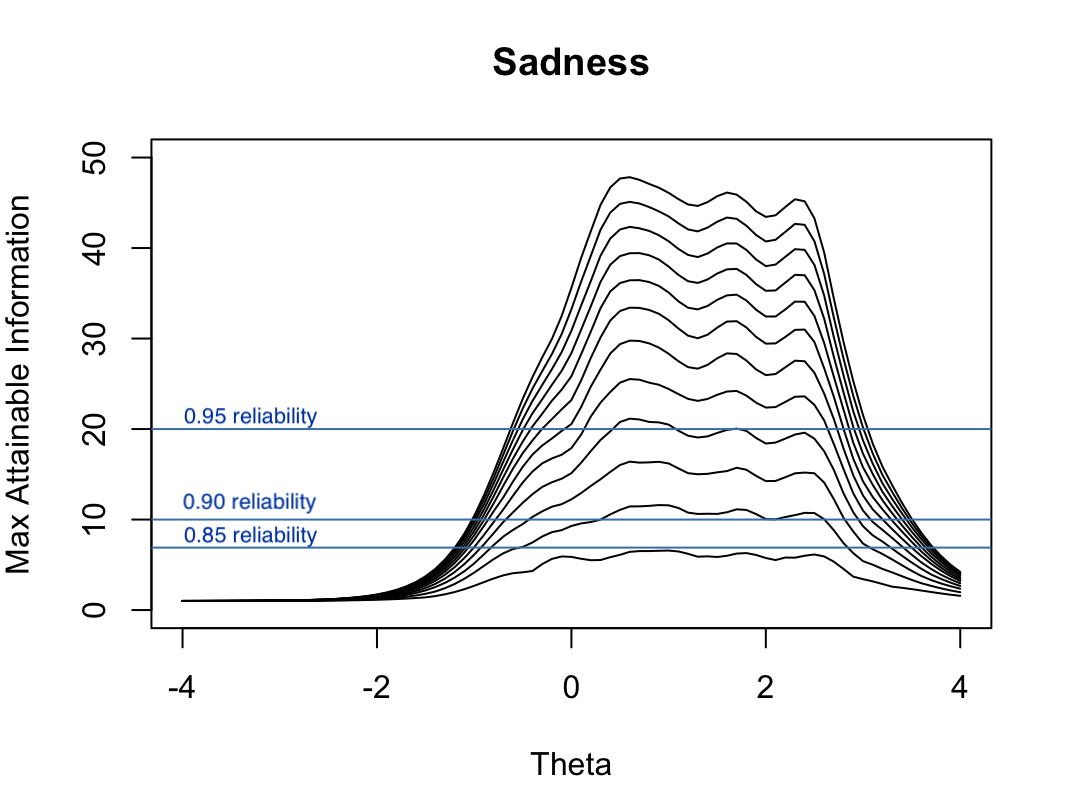 |

Note: The three horizontal lines represent the different target reliabilities (0.85, 0.90, and 0.95). The outermost curve represents the item pool information function for a 12-item CAT stopping rule, while the inner curves represent the maximum attainable information, or reliability, obtained from administering an additional item. There are twelve curves because the maximum number of items administered in the original stopping rule is 12 items
